# Extracellular vesicles as novel drug delivery systems to target cancer and other diseases: Recent advancements and future perspectives

**DOI:** 10.12688/f1000research.132186.1

**Published:** 2023-03-23

**Authors:** Divya Ramesh, Shankar Bakkannavar, Vinutha R Bhat, Krishna Sharan

**Affiliations:** 1 Forensic Medicine and Toxicology, Katsurba Medical College, Manipal, Manipal Academy of Higher Education, MAHE, Manipal, Karnataka, 576104, India; 2 Biochemistry, Katsurba Medical College, Manipal, Manipal Academy of Higher Education, MAHE, Manipal, Karnataka, 576104, India; 3 Radiotherapy Oncology, Katsurba Medical College, Manipal, Manipal Academy of Higher Education, MAHE, Manipal, Karnataka, 576104, India

**Keywords:** EXTRACELLULAR VESICLES, ; Exosomes; Signalling cascade; Biocompatibility; Biogenesis;Cancer

## Abstract

Extracellular vesicles (EVs) are lipid-bound vesicles produced into the extracellular space by cells. Apoptotic bodies (ApoBD), microvesicles (MVs), and exosomes are examples of EVs, which act as essential regulators in cell-cell communication in both normal and diseased conditions. Natural cargo molecules such as miRNA, messenger RNA, and proteins are carried by *EVs* and transferred to nearby cells or distant cells through the process of circulation. Different signalling cascades are then influenced by these functionally active molecules. The information to be delivered to the target cells depends on the substances within the *EVs* that also includes synthesis method. *EVs* have attracted interest as potential delivery vehicles for therapies due to their features such as improved circulation stability, biocompatibility, reduced immunogenicity, and toxicity. Therefore, *EVs* are being regarded as potent carriers of therapeutics that can be used as a therapeutic agent for diseases like cancer. This review focuses on the exosome-mediated drug delivery to cancer cells and the advantages and challenges of using exosomes as a carrier molecule.

## Introduction

In multicellular organisms, interaction among the cells is required to sustain cellular activities and tissue homeostasis. Direct contact between the cells, are used to facilitate intercellular communication. ^1^ According to Wolf *et al*., 1967, microvesicles, formerly known as “platelet dust,” were initially recognised as a subcellular substance derived from thrombocytes that was present in both blood serum and plasma. These extracellular vesicles (EVs) possess importance in communication between the cells. These functions are done by transporting membrane and cytosolic proteins, lipids, and RNA between cells. ^1^ Exosomes are a type of extracellular vesicles with a diameter of 40–150 nm that are produced by all cells and exchanged between them. ^2^ Other vesicles engaged in intercellular communication include MVs, ectosomes, shedding vesicles, and microparticles. ^3^^–^^5^ Exosomes are produced from various cells when multivesicular bodies merge with the plasma membrane. ^6^^,^^7^ Natural sources of exosomes are saliva, blood, semen, urine, plasma, cerebrospinal fluid, and breast milk. ^8^^,^^9^ Exosomes are typically distinguished by their size, as well as the expression of exosome marker proteins, such as CD63, CD81, and CD9, *etc.* ^10^^–^^12^ EVs have been studied for their capacity to deliver drug to treat conditions like cancer and other diseases such as liver ailments, ^13^ nerve disorders, ^14^ sepsis treatment, ^15^ and wound healing. ^16^

### Classification and composition of extracellular vesicles

**Figure 1.**
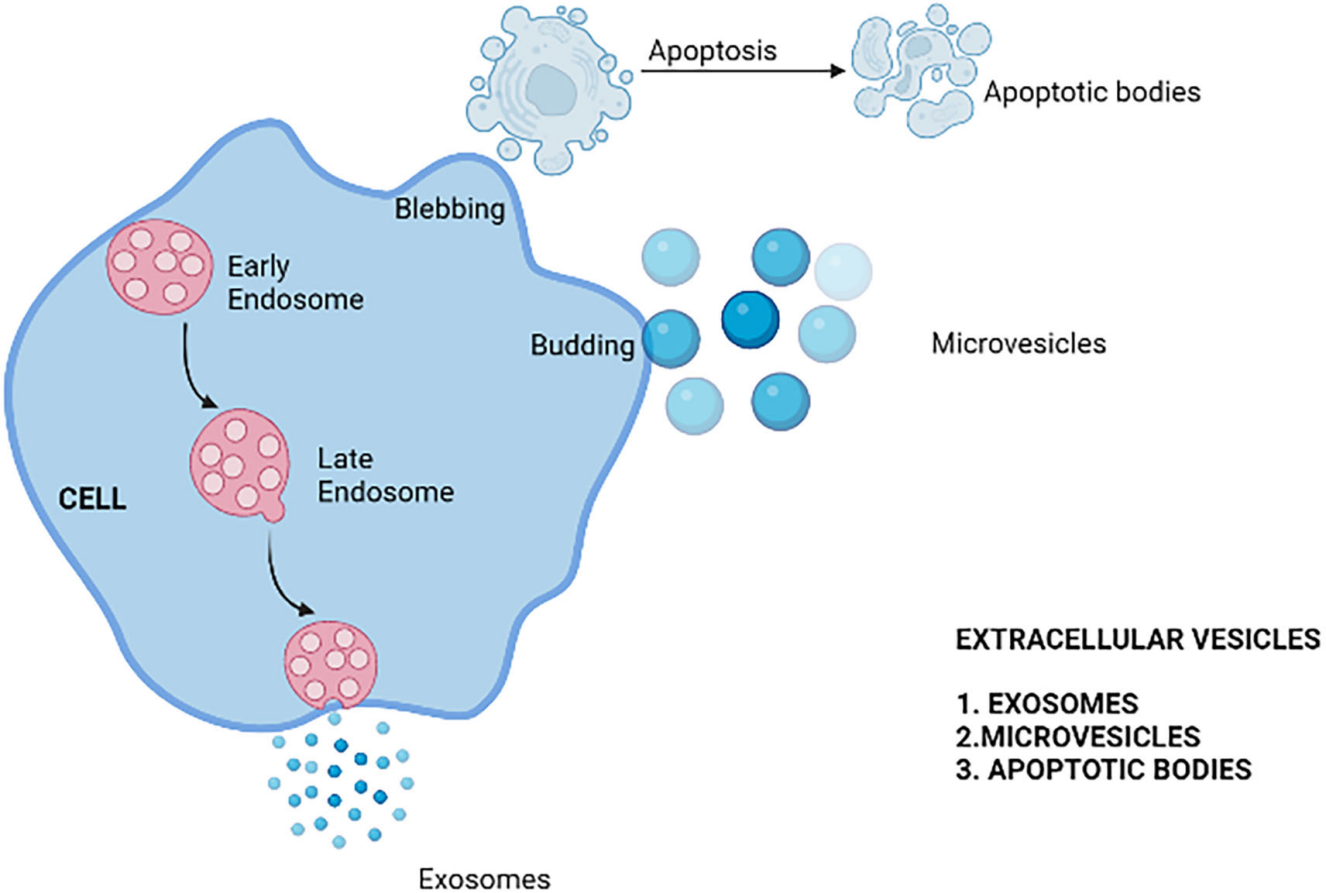
Diagram illustrating the classification of extracellular vesicles.

There are three types of EVs, namely Exosomes, microvesicles, and apoptotic bodies. These are differentiated and classified according to size and method of biogenesis ^17^^,^^18^^–^^21^ as depicted in Figure 1. Exosomes are endocytic in origin formed by plasma membrane budding and blebbing. ^22^ The density, content, and function of EVs are further distinguished. ^22^^,^^23^ The details of various vesicle subtypes are as shown in Table 1.

**Table 1.** Table depicting various subtypes of extracellular vesicles.

Parameters	Exosomes	Microvesicles	Apoptotic bodies
Size	40–150 nm ^24^	50–1000 nm ^25^	500–2000 nm
Density	1.13–1.19 g/mL ^24^	1.04–1.07 g/mL ^25^	1.16–1.28 g/mL
Origin	Endocytic ^18^	Budding from plasma membrane ^17^	Blebbing of plasma membrane ^17^
Composition	Higher level of ceramide compared to plasma membrane ^26^	Similar distribution of the lipids like that of plasma membrane. The difference lies in even distribution of major membrane lipids such as phosphatidylserine, phosphatidyl ethanolamine *etc.*: ^25^	Presence of phosphatidylserine on the cell surface (externalization) along with Annexin, FasL/FasR, TNFα/TNFR1Apo2L/DR5 ^21^
Contents	MHC, miRNA, Non-coding RNA ^27^	Cytoplasmic protein, Membrane proteins, Receptor proteins, miRNA, mRNA ^28^	Various cell organelles, DNA, Nuclear fractions. ^29^
Biomarkers	TSPAN29, TSPAN30, CD81, CD82, CD9 TSG101, CD63 ^30^	Integrins, selectins, metalloproteinase ^31^	Annexin V positive and phospatidylserine (PS) ^32^

### Exosomes

**Exosomes** are specific type of EVs that resembles structures of cells. ^33^ They are now recognised for playing significant roles in cellular communication through various methods. Cell–cell communication occurs *via* the delivery of peptides, metabolites, and nucleic acids to targeted cells.

Exosomes have certain biological properties like immune response, ^34^ repairing tissues, ^35^^,^^36^ stem cell maintenance, ^37^ *etc.* Biological properties of exosomes such as immune response regulation is due to the secretion of the extracellular vesicles from the dendritic cells (DC). This process helps in the transfer of peptides like major histocompatibility complex class II (MHC II) to other DC. Membrane proteins are also transferred intracellularly during the interaction between CD4+ and CD8+ cells. Exosomes derived from DCs have been found to be distinct intercellular vectors. Hence DC-derived exosomes aid in the regulation and maintenance of the immune response. Exosomes also help in the process of tissue repair after an injury. The process of tissue repair has been observed in the exosomes that are derived from mesenchymal stem cells (MSC-Ex). Mesenchymal stem cells derived from the human umbilical cord (HucMSC-Ex) prevent apoptosis and accelerate the process of healing by promoting the cell proliferation. Re-epithelialization of the cells during wound healing is due for the activation of Wnt/β-catenin derived from the HucMSC-Ex. During this process there will be an increased expression of proteins such as collagen I, PCNA (proliferative Cell Nuclear Antigen) and CK 19 (Cytokeratin 19) respectively that promotes wound healing and tissue repair. HucMSC-Ex are found to cure renal tube injuries and has a function in escalating the cell–cell communication. ^38^ Treatment of a wound with HucMSC-Ex reverses AKT (AK Strain Transforming) inhibition and promotes proliferation of the cells thus helping in the healing process. Cell–cell communication by the exosomes also helps in maintenance of pluripotency in neighbouring cells including the embryonic stem cells (ES Cells). ES cells derived from the membrane vesicles or exosomes tend to express proteins responsible for the self-renewal and maintenance of stem cells and its pluripotency. Maintenance of pluripotency will be done by enhanced expression of proteins such as Oct-4, Nanog and Rex 1 respectively. Overexpression of Wnt along with these proteins also enhances the pluripotency of cells thus helps in maintaining the same throughout the generations. These vesicles are involved in various clinical applications such as carriers used to transport cargo for therapeutic purposes. ^39^ These vesicles are preferred due to their reduced immunogenicity and structure. The structure of exosomes includes the closed lipid bilayer thus protecting them from the degradation of cargo. This membrane composition helps these to cross the membranes of the brain including the blood–brain barrier easily and freely compared to other carrier substances. ^40^^,^^41^ Even though all these advantages make the exosomes an excellent vehicle for cargo transport, the major disadvantage is difficulty in targeting specific organs by reducing the off-target distribution. ^41^^–^^43^

Off target distribution of the exosomes is a major challenge in using exosomes as a delivery vehicle. To override this problem the exosomes can be surface modified easily by using chemical modification and genetic engineering. In the process of genetic engineering, the gene sequence of the guiding polypeptide will be fused with membrane proteins of the exosomes. This method is best suited for the surface display of only the membrane proteins on the exosomes. A wide range of surface modification can be achieved through chemical modification strategies. ^44^ This method facilitates display of wide range of both natural and artificial ligands on the exosomes by assembly of fats. Reactions through conjugation will modify the exosomes in a stable manner, but the efficiency of this reaction will be reduced due to the intricacy of the proteins on the surface of exosomes. Assembly of the lipids on the exosomal membranes will cause exosome toxicity. Compared to the method of chemical modification, genetic engineering is more favoured due to its high advantages. It is the most convenient method to impose changes on the exosomes. Genetic engineering is done by the fusion of peptides or ligands of interest on the transmembrane proteins of the exosomes followed by transfection of the donor cells with an engineered plasmid, which facilitates the release of modified exosomes to the target cells or tissues. The lysosome-associated membrane protein (LAMP) family that includes LAMP-2B is currently the major exosomal surface protein used. LAMP-2B family is localized to endosomes and lysosomes with a very small fraction expressed on the exosomal surface (only N terminus). ^45^ LAMP-2B has been used in targeting various tissues, for example, the RVG (rabies virus glycoprotein) peptide of rabies virus fused to LAMP-2B was effectively delivered to acetylcholine receptors and hence delivered drugs to specific areas of the brain. This has been used in targeted therapy of Alzheimer’s disease and it also helps in neurogenesis. ^46^ LAMP-2B was also used in another experiment to deliver exosomes loaded with doxorubicin to target breast cancer cells through intravenous injection. Besides LAMP-2B, the tetraspanin superfamily CD63/CD9/CD81 are also used in exosome engineering. All these protein families can be used in surface modification and targeting; hence these can be used to prevent off-targeting.

**Figure 2.**
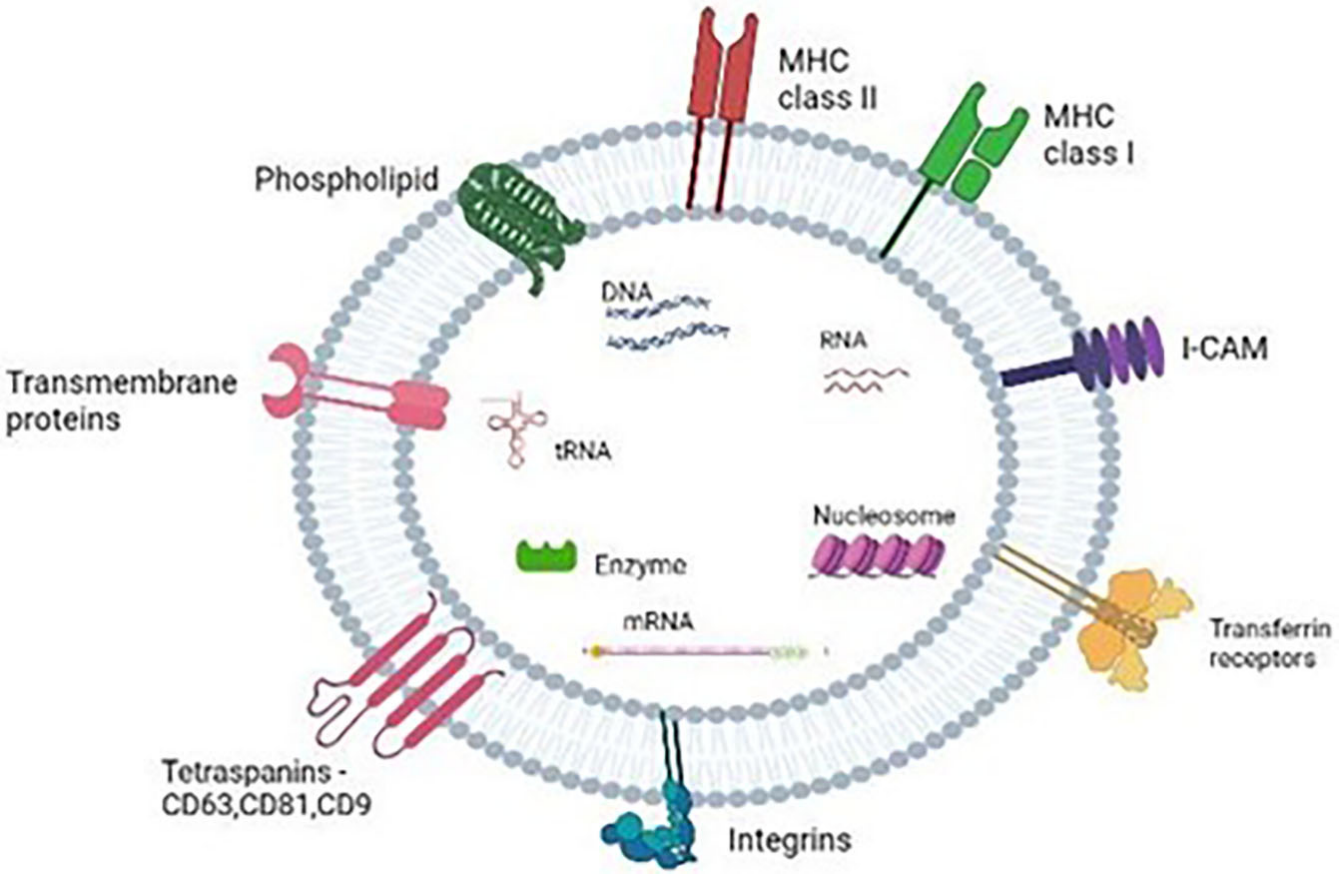
Diagram illustrating the composition of exosomes.

Exosomes carries different types of cargoes that includes proteins, amino acids, lipid and its derivatives and various metabolites. ^47^ Shape of exosomes varies from spherical shape when it is in solution, but it changes to biconcave, or cup-shaped during the phases of processing. ^48^ The major cargo proteins include the tetraspanin class of proteins such as CD9, CD63, CD53, *etc.*, that helps in targeting, ^49^ ESCRT (Endosomal Sorting Complexes Repaired for Transport) proteins required an endosomal sorting complex is essential for the translocation, certain other peptides such as integrins, heat shock proteins (HSP), *etc.* ^50^ as shown in Figure 2. Other than the common protein cargoes cell-specific peptides such as Major Histocompatibility Complex (MHC) class I and MHC class II depend on type of donor cells. ^51^

Exosomes also contain cluster of nucleic acids such as deoxyribonucleic acid, ribonucleic acid, non-coding RNA and micro RNAs (miRs) make up one of the abundant types of RNAs in the exosome. ^52^ The exosomal membrane also consists of many lipid derivatives such as ceramide, and sphingomyelin that help to sort cargoes, transportation, secretion, *etc.*, ribosomal RNA (rRNA), long non-coding RNA (lncRNA), transfer RNA (tRNA), small nuclear RNA (snRNA), small nucleolar RNA, and p-element-induced wimpy testis (piwi)-interacting RNA are some of the other exosomal RNA species that have an impact on biological processes, particularly in tumour development. ^39^^,^^53^

### Microvesicles

Microvesicles (ectosomes or microparticles) are a type of EV that gets produced from the cell membranes. ^54^ In eukaryotes and other multicellular organisms microvesicles are also found in body fluids and tissues along with the other extracellular vesicles. Size of the microvesicles ranges from 30 nm to 1000 nm. ^55^ Microvesicles function similar to other extracellular vesicles in the process, such as tumour immune supression, metastasis, angiogenesis *etc.* ^56^ Microvesicles also helps in the removal of proteins misfolded during protein translocation and it helps in the removal of cell wastes or cell debris from the cellular microenvironment. ^57^ Cancer growth and metastasis can be determined from the level of microvesicles in the body. Hence microvesicles can also be used as biomarkers.

Large number of molecules are present inside the microvesicles. Microvesicles do not contain organelles like that of the cells. It is devoid of nucleus, golgi, endoplasmic reticulum *etc.* Hence microvesicles are not regarded as the intact cells. ^58^^,^^59^ Membranes of the microvesicles are made of membrane lipids as well as proteins. Main function of all the microvesicles is membrane transport and fusion. The membrane is made of phopholipid bilayer and also houses different kinds of proteins. ^58^

Some proteins are specific to the origin while some are common to all the microvesicles. Some tetraspanin family of proteins such as CD9, CD37, and CD63 can be seen on the surface of microvesicles. ^58^^–^^61^ These surface markers helps in the protein sorting and selection of specific cargoes into lumen of microvesicles or membrane. ^62^ In addition to lipids ad peptides microvesicles also contains some nucleic acid such as messenger RNA ( mRNA) and microRNA ( miRNA). ^61^

### Apoptotic bodies

Cells undergoing the process of apoptosis release certain extracellular vesicles called as apoptotic bodies. Its size ranges from 500 nm–2 μm and they are more in number compared to other extracellular vesicles. Appearance of apoptotic bodies is an indicator of cell death or apoptosis while the other extracellular vesicles such as exosomes and microvesicles plays a major role in intercellular trafficking. ^63^ A single cell may produce large number of apoptotic bodies during the apoptosis. Apoptotic bodies (ApoBDs) varies in the content as well as in the size. ^64^ The contents within the apoptotic bodies varies accordingly such as chromatin remnants, some parts of chromatin, some organelles or portions of it. ^65^ Apoptotic bodies are one of the least studied extracellular vesicles. Apoptotic bodies forms during the process of apoptosis and large number of apoptotic bodies will be released during the death of a single cell. Apoptotic bodies are engulfed by macrophages or cancer cells after being released in extracellular space and are then broken down in phagolysosomes. “Tingible body macrophages” are macrophages that consume apoptotic cells. ^66^^,^^67^

Removal of apoptotic bodies doesn’t produce any inflammatory reactions within the body as there is no release of any free contents or cellular debris to the extracellular environment. Once the ApoBDs are produced they will be engulfed by phagocytes thus preventing the process of necrosis. The phagocytes after the engulfment doesn’t produce certain inflammatory cytokines thus preventing any complications in the body during the apoptosis and formation of ApoBDs. ^68^^,^^69^

### Biodistribution of extracellular vesicles

Cells like dendritic cells, stem cells, macrophages, *etc.* will secrete exosomes. ^70^ Exosomes are widely distributed and found in various body fluids such as synovial fluid, breast milk, urine, blood plasma, saliva, and amniotic fluid. ^71^ The biodistribution of exosomes is dependent on the mode of administration of the same. ^70^ The administration through the intravenous route results in biodistribution of the exosomes to various organs such as the liver, kidney, lungs, and spleen, ^70^ and this intravenous injection of exosomes will result in rapid clearance from the bloodstream, but other methods like intranasal allows the fast transport to the brain. ^40^^,^^72^ Therefore, exosomes are believed to be used as one of the best vehicles for targeted drug delivery. The size of exosomes also matters when targeting specific locations as the larger exosomes will get trapped in lymph nodes, bones as well as in liver. ^73^ Targeting the exosomes to a specific area is important for the delivery of exosome contents to the target. Targeted delivery is often dependent on the markers present on surface of exosomes. ^74^

Although circulating microparticles are seen within blood of the individuals without any disease conditions. The levels of microvesicles drastically increase during certain diseases like hypertension, cardiovascular diseases ^75^ and pre-eclampsia ^76^ *etc.* In some instances the surface molecules of the endothelial micoparticles change, thus it can be regarded as the indicator of various diseases. Microvesicles are shed into the outer environment by membrane fission by formation of invaginating bodies to the outer environment. Budding of these extracellular vesicles depends on the specific locations depending on the type of proteins and lipids incorporated. Microvesicles are released at the specific location after the cargo loading. ^77^

Apoptotic bodies are a special types of extracellular vesicles that are released from the cell that is undergoing apoptosis. ApoBDs are specifically formed from the plasma membrane through the process of membrane blebbing. These structures are found throughout the body during the apoptosis and it is also a carrier of DNA, RNA, and lipids *etc.* and are considered as tiny sealed pouches. ^78^

### Transport of extracellular vesicles and reaching target sites

Once extracellular vesicles has been released from the donor cells or tissues, it will interact with the target tissues through endocytosis. Once the EVs has entered, it releases cargo into the target cells. ^79^

Exosomes on reaching the target sites interacts with receptors on cell membrane or it will be internalized. The cell surface peptides bind with the surface ligands of EV and trigger a cascade of reactions to activate the target cells. This process is used in the process of immunomodulation and apoptosis respectively. ^80^

**Figure 3.**
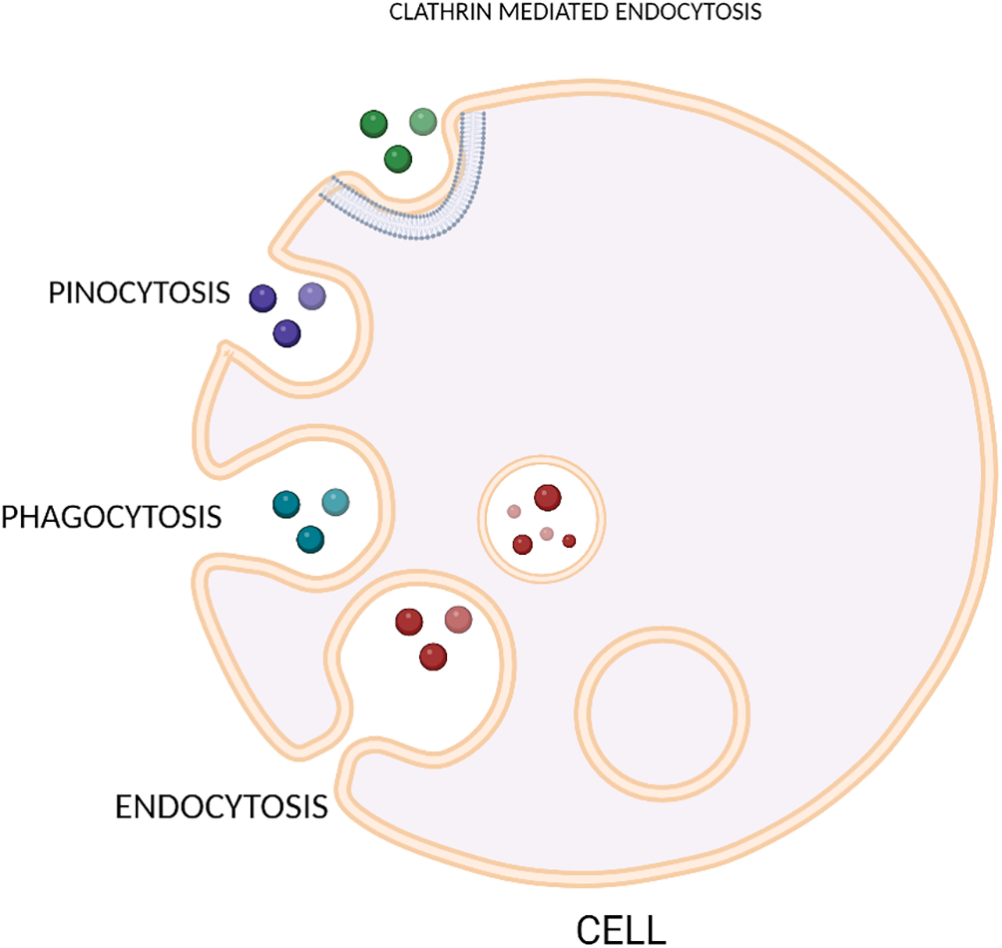
Diagram illustrating methods of EV uptake by the cells.

EV uptake can also be done through the mechanism of membrane fusion, where the fusion of two hydrophobic membranes occurs. During this process, EVs interact with cells through plasma membrane fusion and content release. Fusion of membranes are employed by SNARE (Soluble N-Ethylmaleimide Sensitive Factor Activating Protein receptor) and RAB (Ras- Associated Binding) proteins ^81^^,^^82^ The various methods through which EVs are taken up by the cells is depicted in Figure 3.

Another method by which exosome components are delivered to target cells is through internalization process. Endocytic pathways are used in the process of internalization where the contents of the exosomes are delivered to target cells. ^83^^–^^85^ The process of cellular uptake is temperature sensitive and is directly proportional to the temperature fluctuations.

Endocytosis mediated by clathrin molecules is characterized by the involvement of receptors and ligands. This method of internalization occurs in various types of cells like ovarian and colon tumour cells, ^74^^,^^86^^,^^87^ cardiomyocytes, ^88^ hepatocytes, ^89^ macrophages, ^89^^,^^90^ *etc.* are intenalized by clathrin protein. Dynamin-2 protein plays an important role in clathrin-mediated endocytosis and helps in the scission of the vesicle by forming a collar shaped structure in the neck of invagination. Inhibition of this protein shows reduced exosome uptake by macrophages. ^90^^–^^92^ In certain cancer cell types, exosome uptake is enhanced by a transferrin receptor protein. This exosome uptake pathway is mediated by exosome and cargo composition. Caveolin dependent endocytosis (CDE) also has an important function in internalization. In CDE, plasma membrane forms tiny, cave-like invaginations called caveolar vesicles that are eventually torn off and absorbed into the cell. The cell membrane contains caveolin that forms glycolipid rafts like cholesterol, sphingolipid. Caveolin proteins help in the formation of caveolae and for the generation of caveolin rich rafts caveolin has to be oligomerized. ^93^ A particular type of caveolin protein called caveolin 1 is considered as important for the EV uptake ^94^ and its inactivation resulted in defective uptake or no uptake.

Ruffled extensions of the plasma membrane during macropinocytosis or cell drinking causes the internilization of EVs thus releasing the cargo into specific locations. For the process of macropinocytosis to occur, it requires the activation of Rac1 GTPase for the invagination and also it requires proper functioning of Na ^+^/H ^+^ exchanger and cholesterol. ^95^^,^^96^ It has been observed that impairment of the same results in the failure of EV uptake.

Phagocytosis is also considered as one of the methods by which EV uptake occurs into the cells. Phagocytosis or “cell eating” is opposite to macropinocytosis or “cell drinking”. Unlike pinocytosis, this process does not require ruffles of the membrane for the engulfment of EVs, instead it involves the formation of membrane invaginations. Compared to other modes of EV uptake, phagocytosis is regarded as the most effective. ^97^

### Biological functions of extracellular vesicles

EVs secreted from various types of cells through different mechanisms has proved to be biologically important. Extracellular vesicles are most popularly known as the carrier vehicles for most of the cargoes such as nucleic acids, drugs,proteins *etc*; and it delivers these substances to the specific receptor cells. Hence these nanocarriers are regarded as the most effective vehicles for the transportation of biological agents. It has been observed that the function of EVs depend upon the type of cargoes they carry. EVs carrying certain RNAs or long non coding RNAs help in the development of certain types of cancers. ^98^ Even though some studies proved that cargo carried by EVs results in cancer development, some studies have proved that these cargo loaded EVs help in treating cancer. ^99^ Function of EVs also depends on the cell of origin. EVs produced from some cancer cells promotes cell communications, thus producing specific signals for apoptosis, cell migration, growth *etc.* ^100^ Thus EVs aid in the determining the fate of certain cells. Function of EVs also depends upon the nature of the cargoes carried by them. In a study conducted by Xue *et al*., 2017, it was explained that EV loaded with circular ribonucleic acid plays a role in the proliferation of Hepatocellular carcinoma cells. ^101^ It was observed that the expression of some biomarkers such as cyclin D1 and EZH2 (Enhancer of Zeste Homolog) were increased rapidly due to the presence of circRASSF2 in the case of laryngeal squamous carcinoma (LSCC). Thus it was confirmed that cargo loaded EVs has the capacity to control the growth of the cells. ^102^ Role of EVs in promoting autophagy and apoptosis has been a milestone in the treatment of cancer. EV loaded with certain anticancerous substances has been proved to promote cell death in HCC as well as in Glioblastoma. ^103^

**Role of extracellular vesicles in pathogenesis**

According to Zitvogel *et al.,* 1998, EVs such as exosomes obtained from the Dendritic cells and can express major histocompatibility complex I. Exosomes are also involved in various disease conditions such as a variety of cardiovascular physiological and pathological conditions. ^104^^,^^105^ Recent research has found exosomes released from mammalian cells with normal physiology contributed to cell–cell communication in cardiac tissues. These vesicles consist of proteins, lipids, and genetic materials. Vesicles released by the cardiac cells consist of APOBDs, MVs and exosomes. ^106^ It has been observed that exosomes are released from different cells within the heart and hence they are involved in various cardiac pathophysiological conditions.

It was noted that EVs play a major role in development of atherosclerosis. Atherosclerosis mainly occurs due to endothelial dysfunction due to impediment in the normal blood flow through the endothelium. Endothelial dysfunction occurs due to the increased secretion of oxidized LDL and homocysteine in the serum. According to Tsuda, 2015, ox-LDL and Hcy release the HSP70-containing exosomes from cultured aortic ECs which in turn induces the activity of monocytes through the proinflammatory differentiation of macrophages, further resulting in the development of atherosclerosis. ^107^ Once atherosclerosis has formed, the blood vessels will be narrowed due to subsequent accumulation of plaque over years. The plaques once formed lead to irregular blood flow, leading to blood clots, and finally complete occlusion of the artery, finally leading to myocardial ischaemic injury. Myocardial ischaemic injury is caused due to alterations in the circulation of micro-RNA and the majority of circulating miRs are found enclosed within the EVs. Under any conditions of stress, fibroblasts promote the pathological hypertrophy of cardiac cells through exosomes and their micro-RNA load. In peripartum cardiomyopathy (PPCM), the elevation of miR-146a in endothelial cells blocks the blood vessel formation and increased release of endothelial cells derived miR-146a-enriched exosomes. The EVs once released from the endothelial cardiomyocytes results in the advancement of levels of miR-146a. This process will cause reduced expression of miR-146a target proteins like Erbb4, Notch1, and Irak1 in cardiomyocytes that causes decreased metabolic function. From these findings, it can be inferred the miRNA-based cell interaction of endothelial cells and cardiac muscle cells involves EVs, and hence EVs are also responsible for various cardiac pathologies.

EVs has a role in the pathogenesis of neurodegenerative disorders. These disorders include the deposition of a specific protein in neuroanatomical locations. It is proved that major proteins involved in the pathology of these diseases are transported by the exosomes. The cerebrospinal fluid and blood of the neurodegenerative diseases affected individuals contain exosomes loaded with aggregated proteins that are specific to these diseases. Moreover, exosomes provide a favourable condition for protein transport into the brain. ^108^

The role of EVs in cancer involves the transfer of cancer-causing proteins and nucleic acids that mediates the activities of the cells like its role in tumourigenesis. ^109^ EVs obtained from the tumour cells facilitate tumour angiogenesis by converting the fibroblast and mesenchymal cells into myofibroblast. EVs isolated from the tumours can move neutrophils and skew the M2 polarization of the macrophages that helps in promoting the metastasis of the tumours. These exosomes also help in the development of multidrug resistance to the tumour cells by transferring certain MDR proteins and miRNAs as well as exporting drugs against and by neutralizing antibody-based drugs. ^113^ Extracellular vesicles can thus play a dual function in cancer regulation, preventing or boosting growth, based on their bioactive payload and cell from where it originated.

### Role of extracellular vesicles in the cancer treatment

Since it has been proved that EVs unload their contents into the target cells, it has developed attraction in the field of oncotherapy. ^110^^–^^112^ Various methods are being used in recent times to cure cancer using extracellular vesicles as described below:

**Using EVs derived from the immune cells to suppress the growth of cancer cells**

In cancer immunity, the dendritic cells are involved in the inhibition of tumour growth and metastasis, and this is done by capturing the new antigens or foreign bodies and hence producing the marked antibody response that involves cytotoxicity to specific tumour. ^113^ EVs derived from the dendritic cells contain specific cargoes that are responsible for the antigen presentation and hence it acts as a perfect treatment for cancer. ^114^ Zitvogel *et al.,* 1998, ^115^ suggested that tumour peptide-pulsed dendritic cell-derived exosomes could activate the cytotoxic T cells-based activity to reduce the metastatic growth in mouse that occurs within the body. In a study done by Munich *et al*., 2012, it was observed that dendritic cells kill the tumour cells and activate the natural killer cells through the TNF family ligands. ^116^

**Inhibition of cancer derived EVs release**

EVs derived from the cancer cells cannot be used for the treatment of cancer as it will accelerate the growth of cancer by providing favourable conditions for metastasis, immune escape, angiogenesis, drug resistance, *etc.* ^89^^,^^90^ Therefore, the effective treatment of cancer can be achieved by inhibiting the synthesis, release, and uptake of the cancer derived EVs. ^117^ Another technique to slow tumour growth is to use a haemofiltration system to remove circulating EVs. Tumours secrete a large number of EVs such as exosomes into the extracellular space, and it carries oncoproteins, immune suppressive molecules that help in tumour progression and metastasis. Several strategies have been proposed for targeting the malignant activities of tumours. Haemofiltration of the EVs is done to remove the circulating EVs from the blood. It is done using an affinity plasmapheresis platform known as adaptive dialysis-like affinity platform technology (ADAPT TM) system that helps to override the toxicity risks interactions of the drugs. This technique uses certain affinity agents that includes lectins and antibodies binding to EVs. These agents will be immobilized in the outer capillary space of plasma filtration membranes. This setup is like existing kidney dialysis system. Working principle for this setup is, when the patient’s blood passes through this device, the plasma components that are <200 nm will move through the porous fibres through the capillary action to interact with the substances that are immobilized on the device. The target molecules will bind tightly to the agent while other components of the blood will pass through the membrane. This device is multifaceted since large number of antibodies and other high affinity substances such as ligands can be absorbed into the cartridges to capture single or multiple targets. Although ADAPTTM therapies necessitate vascular surgery for patients, this subtractive approach to treat cancer using exosomes would avoid drug toxicity or any interaction hazards, thus giving it an edge over other pharmacological methods. As a result, the concept of this device offers a method for targeting exosomes that needs to be investigated for its potential as an additional therapeutic candidate to current cancer therapy protocols. This alternative could also be used as a therapeutic option for curing immunological dysfunction and improving immune responses to tumour growth. ^118^

**EVs as carriers of genes**

EVs are being regarded as the effective carriers of the gene, but the usage of exosomes derived from the natural sources are difficult to study and expected therapeutic effects are achieved very rarely. The EVs that are modified through genetic engineering are more effective against cancer. EVs can also be used as miRNA carriers for effective treatment against cancer. ^119^ miRNAs are tiny noncoding RNAs, and these are small and endogenous in nature. These miRNAs are used as an effective tool for cancer therapy. The major drawback of these miRNA mediated delivery is that miRNAs molecules get destroyed quickly in *in vivo* conditions. Therefore, the delivery of miRNA to specific location is considered as a difficult task. But if these miRNAs are encapsulated within the EVs, these can be delivered at the target site. ^120^^–^^124^ EVs enriched with the anti-glioma miRNA (miRNA-146b) can decrease growth of glioma in the laboratory conditions according to Katakowski *et al*., 2013. ^125^ EVs enriched in miR-101 can also inhibit osteosarcoma cell invasion/migration *in vitro* and metastasis *in vivo.* ^46^ EVs are also being used as protein carriers for cancer therapy, where these can be loaded with tumour antigens, ^126^ proteins that induce apoptosis, ^127^ mutant and deficient proteins in apoptosis, several nanobodies, ^128^ transferrin, and lactoferrins, ^129^ proteasomes ^129^ *etc.* for targeted therapy. Dendritic cells are used for T cell mediated immunotherapy by presenting the antigen from tumour to naïve T cells. But due to the low life span of DCs its use is limited. ^130^ When compared to antigen presenting DCs, the exosomes isolated from the dendritic cells consists of complexes made from major histocompatibility factors and peptides and some co-stimulatory molecules on their membrane, that allows them to continue with the antigen presentation and enhance the process of immunisation in murine models. ^131^ Survivin, an anti-apoptotic protein, suppresses the activation of cell death in large number of cancer cells.

According to the studies conducted by Aspe and wall in 2010, it was observed that Survivin-T34A has an inactive mutation that reduces its pro-survival function that causes caspase activation followed by death of tumour cells. ^132^

**EVs as carriers of chemotherapeutic drugs**

Anti-tumour chemotherapeutic medicines can successfully kill tumour cells that are rapidly developing. However, these medications have the potential to harm normal, healthy, fast-growing cells, resulting in major side effects. Furthermore, it is difficult for some hydrophobic medicines to target tumour cells with specificity. As a result, these formulations need more specific and efficient carrier. EVs have the capacity to be an efficient carrier for chemotherapeutic drugs as a result of their stable lipid bilayer structure. ^119^ Chemotherapeutic drug-loaded exosomes particularly targeting cancer stem cells *in vivo* are the best exosome cancer therapy. Exosome-mediated chemotherapeutic administration has been proven to have significantly better anti-tumour effects in animal tumour models than free medicines. Doxorubicin, for example, is a frequent chemotherapy medication to treat haematolog ical cancers and a variety of solid tumours. ^133^ Paclitaxel is another antimitotic chemotherapeutic medication that is commonly used in cancer treatment. ^134^^,^^135^ Sonication can be used to load paclitaxel into exosomes, and it has proved to be 50 times more cytotoxic than the free paclitaxel in drug-resistant cancer cells *in vitro.* In a rat model of Lewis lung carcinoma, they can also significantly reduce tumour size and stop pulmonary metastasis. ^136^ This suggests that paclitaxel contained in exosomes can target drug-resistant CSCs directly. Furthermore, paclitaxel-loaded exosomes from prostate cancer cells had increased cytotoxicity in autologous cancer cells. ^137^ Drug-pre-treated donor cells can also create drug-loaded exosomes, which is interesting. EVs produced from mesenchymal stem cells treated with paclitaxel, for example, had a significant suppressing activity on tumour cells of pancreas. ^138^

### Role of extracellular vesicles in treatment of other diseases

Even though extracellular vesicles contribute to the pathology of some diseases it is also used as a therapeutic agent against certain diseases other than cancer. EVs loaded with cargo have proved to be effective against some diseases. Upadhya and Shetty, 2021, observed the therapeutic effects of EVs on the Parkinsons disease model (PD). It was studied that MSC -derived EVs protect dopaminergic neurons thus reducing apoptosis. This leads to the improved motor function in the Parkinson disease models. Hence it has been proved that EVs are effective against neurodegenerative diseases. ^139^

EVs also contributes to the protection of cardiac cells thus acting as a cardioprotective agent. EVs obtained from stem cells provide cardio protection, thus acting as a therapeutic tool against cardiovascular diseases. Stem cell-derived preconditioned cardioprotective exosomes are believed to be involved in cardio protective mechanisms. Hence Exosomes play an important role in cardioprotective as well as pathological conditions of heart cells. ^105^

EVs are also known to provide protection against inflammatory bowel disease (IBD) produced from dextran sodium sulfate through intestinal stem cell and epithelial regeneration. It was observed that EVs derived from the MSC-derived EVs resulted in normal functioning by downregulating inflammatory responses, thus promoting the regeneration of epithelial cells or tissues. Hence EVs can be used as a medicine for treating IBD. ^140^

### Challenges of extracellular vesicles in drug delivery

There are lots of challenges in using EVs as a delivery vehicle. Firstly there are no effective methods for isolation of EVs from various sources. Nowadays EVs are isolated using chemicals such as polyethylene glycol (PEG) which provides low specificity but high recovery. EVs isolated using ultracentrifugation method, size-based isolation techniques; immunoaffinity capture-based techniques; precipitation; microfluidics-based isolation technique provides medium recovery and specificity. Immunoaffinity based techniques and isolation using microfluidics are considered as low recovery with high specificity. Hence there is a requirement of high yielding, more economical and time saving method for the isolation. ^141^

Second challenge is limited drug loading efficiency of EVs compared to other nanoparticles like liposomes. The reduced efficiency causes difficulty in loading exogenous drug into the EVs thus making it a great barrier in using the same for other applications. This may be due to its small size and reduced capacity compared to liposomes or other nanovesicles. This can be resolved by using efficient drug loading strategies like transfection, co-incubation for longer time, electroporation, rapid freeze thaw cycles. ^141^

The third major challenge is instability of EVs when stored for longer time. It has been observed that EVs that are stored for longer time in −80°C loses its viability and efficiency within 2 weeks. Hence, there is a requirement of more efficient storage method that will preserve EVs for longer duration without degrading it. ^142^

## Conclusion and future perspectives

EVs, as a natural carrier, is developing as a promising agent in cancer treatment. EV based cancer therapies include employing naturally produced immune cell exosomes to reduce tumour cells, blocking cancer cell-derived EV activity, and exploiting EVs as cargo carriers. Using EVs as a potent carrier protein involves lots of advantages as well as challenges. The advantages are EVs are less toxic and immunogenic than synthesized nanoparticles because they are more biocompatible and biodegradable. ^143^ Although other extracellular vesicles obtained from different cells are biologically compatible, they are bigger and more ubiquitously distributed than exosomes, which limits their use in loading and distribution of drug. EVs are very simple to produce because most cell types can manufacture them. Additionally, EVs are present in almost all the body fluids, and their small size allows them to bypass lung clearance and cross the blood–brain barrier. ^144^^,^^145^ Even though there are lots of advantage of using exosomes as carriers, the challenges are the differentiation between exosomes from various sources is still unclear. The number of EVs required to provide a therapeutic impact may range dramatically amongst malignancies. Tumour scalability and heterogeneity may have an impact on treatment outcomes and many other roles of EVs produced from various sources have not been thoroughly investigated. Furthermore, nothing is known about the exosome modifications that help in providing a high degree of selectivity for specific cancer cells. The storage and stability of EVs are still unknown.

EVs offer novel and important applications in treating cancer and other diseases, despite the fact that there are many more challenges in using exosomes as carrier agents.

## Data Availability

No data are associated with this article.
