# Supplementary material for: Extracellular vesicles as novel drug delivery systems to target cancer and other diseases: Recent advancements and future perspectives
Source: F1000Res. 2023 Mar 23;12:329. [Version 1] doi: 10.12688/f1000research.132186.1 (PMC10589634; doi:10.12688/f1000research.132186.1)
Supplement: Peer review history

# Reviewer report: Reviewer response for version 1

**DOI:** 10.5256/f1000research.145092.r204580

**Published:** 2023-10-20

**Authors:** Charan Kishor Shetty

**Affiliations:** 1 Universiti Sultan Zainal Abidin - UniSZA, Kuala Terengganu, Malaysia

Bakkannavar and co-authors investigated the topic of Extracellular vesicles (EVs) as a novel drug delivery system to target cancer and other diseases. They specifically focused on the hypothesis that EVs are regarded as potent carriers of therapeutics that can be used as a therapeutic agent for diseases like cancer.

 The authors have a very well-organized Introduction by elaborating biological functions of Extracellular vesicles, pathogenesis, and their role in cancer treatment. Moreover, they have described how EVs are derived from immune cells to suppress the growth of cancer cells, inhibition of cancer-derived EVs release, EVs as carriers of genes, and finally EVs as carriers of chemotherapeutic drugs and their use in treatment of other diseases. The article also gives us an insight into the challenges of EVs in drug delivery.

 The manuscript is certainly well-written and attractive. Lastly, I think that this article conveys the message that EVs offer novel and important tools in cancer treatment.

 It would be great if the authors could add any ongoing EV carrier drugs in use or under production.

Is the review written in accessible language?

Yes

Are all factual statements correct and adequately supported by citations?

Yes

Are the conclusions drawn appropriate in the context of the current research literature?

Yes

Is the topic of the review discussed comprehensively in the context of the current literature?

Yes

Reviewer Expertise:

Forensic Pathology

I confirm that I have read this submission and believe that I have an appropriate level of expertise to confirm that it is of an acceptable scientific standard.

## References

[1] : Exosome-mediated siRNA delivery to suppress postoperative breast cancer metastasis. *J Control Release* .2020;318: 10.1016/j.jconrel.2019.12.005 1-15 10.1016/j.jconrel.2019.12.005 31830541

# Reviewer report: Reviewer response for version 1

**DOI:** 10.5256/f1000research.145092.r204660

**Published:** 2023-09-15

**Authors:** Raghavendra Babu Y. P.

**Affiliations:** 1 Department of Forensic Medicine, Koppal Institute of Medical Sciences, Koppal, Karnataka, India

The authors have delved on reviewing the literature with regards to EVs as a novel way of delivering drugs in cancer and other diseases.

 The manuscript is lucidly written with wide range of issues related to EVs and their physiology, utility and probable ways to utilise them as drug delivery systems. The review gives factual information about EVs and delves throughly into the current topic of EVs.

Is the review written in accessible language?

Yes

Are all factual statements correct and adequately supported by citations?

Yes

Are the conclusions drawn appropriate in the context of the current research literature?

Yes

Is the topic of the review discussed comprehensively in the context of the current literature?

Yes

Reviewer Expertise:

i Have been involved in Forensic Medicine teaching since last 18 years

I confirm that I have read this submission and believe that I have an appropriate level of expertise to confirm that it is of an acceptable scientific standard.
